# Consistent condom use and its predictors among female sexual Partners of People who Inject Drugs in Klang Valley, Malaysia

**DOI:** 10.1186/s12889-019-7855-1

**Published:** 2019-11-07

**Authors:** Rosliza Abdul Manaf, Nigel Dickson, Sarah Lovell, Faisal Ibrahim

**Affiliations:** 1 Department of Community Health Faculty of Medicine & Health Sciences, Universiti Putra Malaysia, 43400 Serdang, Selangor Malaysia; 2 Department of Preventive & Social Medicine, Dunedin School of Medicine, University of Otago, P.O. Box 56, Dunedin, 9054 New Zealand; 3 School of Health Sciences, University of Canterbury, Private Bag 4800, Christchurch, 8140 New Zealand

**Keywords:** Condom use, Sexual partners, MWID, Malaysia, HIV risk

## Abstract

**Background:**

Men who inject drugs (MWIDs) comprise the highest percentage of diagnosed HIV cases in Malaysia. Their female partners risk being infected through unprotected sexual contact. This paper reports the prevalence of consistent condom use and its predictors among the wives and regular sexual partners of MWIDs in Klang Valley, Malaysia.

**Methods:**

A cross-sectional study using a self-administered questionnaire was conducted among the wives and regular sexual partners of MWIDs in the study location; 221 women were recruited through respondent-driven sampling. Data were analysed descriptively for the prevalence of consistent condom use, HIV status and HIV risk-related behaviour. Subsequently, simple and multiple logistic regressions were undertaken to identify the predictors of consistent condom use.

**Results:**

The prevalence of consistent condom use among respondents was 19.5%. Slightly more than half (52.5%) of respondents had never used condoms with their partner. Fourteen women (6.3%) reported being HIV positive. While 7.7% had HIV-positive partners, 45.7% were unaware of their partner’s HIV status. Consistent condom use was significantly higher among single women (AOR = 4.95; 95% CI: 2.45, 9.99), women who lived in urban areas (AOR = 2.97; 95% CI: 1.30, 6.78), HIV-positive women (AOR = 3.45; 95% CI: 1.13, 10.5) and women involved in sex work (AOR = 3.55, 95% CI: 1.45, 8.67).

**Conclusions:**

Inconsistent condom use among the majority of female sexual partners of MWIDs underscores the heightened risk faced by these women and calls for alternative prevention methods that women are able to control.

## Introduction

Since the first case was reported in Malaysia in 1986, HIV has predominantly affected men, with the sharing of injecting equipment being the most common mode of transmission [1]. Over recent years, there has been concern over the increasing number of cases of heterosexual transmission of HIV in Malaysia, resulting in the changing gender distribution of the disease, which is currently increasing among women [2]. The percentage of HIV diagnosis among women has escalated from 5% in 1997 to 21% in 2017 [1]. Similar situations have been reported in several other Asian countries [3]. These women are at an increased risk of contracting HIV, mainly through unprotected sexual contact with their infected partners. The risk is also frequently augmented by power imbalances in sexual decision making, which are associated with lower rates of condom use among women than among men [4–6].

HIV prevalence among the Malaysian general population was reported as less than 1%. However, the prevalence remains high among high-risk groups, such as female sex workers (6.3%), men who have sex with men (21.6%), transgender women (10.9%) and people who inject drugs (13.5%) [1, 2]. Among the injection drug users, the majority of whom were men [7], HIV prevalence ranged from 16 to 44%, and three-quarters of those with HIV were infected through injection drug use [8–10]. Behavioural surveys have revealed the risks of onward HIV transmission due to 80% of the injection drug users being sexually active and 58% having multiple sex partners [2, 8, 11]. Approximately 40% of the injection drug users were also involved with sex workers or had sexual contact with other men, which meant a higher risk of contracting and transmitting HIV [3]. As such, people who inject drugs have the potential to act as a bridging population for the HIV epidemic, from the mainly injecting route to heterosexual transmission.

Meta-analyses of condom effectiveness in preventing HIV transmission revealed that consistent condom use between discordant couples has resulted in an overall 80% reduction in transmission risk [12], yet reported condom use among high-risk individuals remains low in many parts of the world [13]. Inconsistent condom use has been noted in previous studies among partners of injection drug users in the USA [14], India [15, 16] and Vietnam [5], where the rate of condom use in the studied populations ranged from 13 to 35%.

While previous studies from various locations in Malaysia reported a low prevalence of condom use among men who injected drugs during their last sexual intercourse (only 14 to 28%), limited local data are available to show the prevalence of condom use with their wives and steady partners [2, 8, 11]. This paper reports the prevalence of consistent condom use and the factors associated with this practice among the wives and sexual partners of men who inject drugs in Klang Valley, one of the regions in Malaysia severely affected by HIV through the sharing of injecting paraphernalia amongst intravenous drug users [2].

## Materials and methods

### Study location

The survey was conducted in Klang Valley, Malaysia, which includes the Federal Territory of Kuala Lumpur and four surrounding districts in the neighbouring state of Selangor (Petaling, Selayang, Hulu Langat and Sepang). These areas were selected for their mixture of urban and sub-urban settings. This is a highly populated area, owing to an active economy and industrial activities, and it is home to approximately 10% (2.74 million out of 28.3 million) of the Malaysian population [17].

### Participant selection criteria and recruitment

The eligible participants were women aged 18 years and above who were in a stable sexual relationship with a man who injected drugs (MWID) for at least six months. To ensure that the men were active injection drug users, recruitment was assisted by several non-governmental organizations outreach workers who were familiar with the population of drug users in the surveyed area. Sample size estimation was undertaken using the STATA Data Analysis and Statistical Software, version 13 [18], to detect a difference of 5% in the prevalence of condom use for each independent variable with 80% power when a type I error rate of 5% was assumed [4, 8, 19], giving an estimated sample size of 220.

Given the difficulties in locating this “hard-to-reach” population, respondent-driven sampling (RDS) was undertaken. In RDS, a “seed” amongst the target population of interest is chosen, who will then begin recruiting his/her colleagues through a specially designed coupon with a tracking system. There is a quota for recruitment that helps to reduce oversampling among subjects with larger personal networks and produces a more heterogeneous group [20]. In this study, twelve men who injected drugs (MWIDs) who were married or had a regular sexual partner were initially identified in each location and recruited as the seed. The men were asked to invite their wife/regular sexual partner to the survey. Then, each MWID was provided with three recruitment coupons to pass to their friends who were active injection drug users and married or with a regular sexual partner. The newly recruited men were asked to invite their wife/regular sexual partner to participate in this study. All MWIDs who came with their wife/regular sexual partner to participate were given the opportunity to be a recruiter. During the data collection, the women were provided with a respondent information sheet and were ensured that their participation was voluntary and that they could choose not to participate at any time.

For the successful recruitment of female respondents, regardless of whether or not the women agreed to participate, the MWIDs received a cash amount of 10 Malaysian ringgit (RM10), which is approximately 2.4 US dollars. This is to prevent the possibility of coercion and to ensure that the women are willing to participate on their own accord. An additional RM10 was given as a secondary incentive to the MWIDs for each successful recruit to compensate them for their time and effort in the recruitment process. The women who participated received RM20 for their participation in the survey. Educational materials on HIV prevention were supplied to the couples, including information where testing and, if needed, treatment could be obtained. The value of the incentive given in this study is considered acceptable and falls short of being coercive.

The recruitment chain continued for six months with a maximum chain length of six, resulting in the collection of 332 names and contact numbers of eligible women. Out of 332 eligible women, only 282 were contactable. Of these women, 50 refused to participate, and two women did not come despite agreeing to do so. Nine questionnaires were incomplete and missing several important data, resulting in 221 responses being analysed. The response rate for this study was 78.4%.

### Study instrument

A self-administered questionnaire was used in the survey. It was developed based on the theory of gender and power framework, which included physical exposure and behavioural risk factors, social norms and culture and socio-economic risk factors [21, 22]. Physical exposure and behavioural risk factors were conceptualized into partner’s HIV status, the use of injectable drugs among the participants and their involvement in sex work. Social norms and culture were conceptualized into marital status and sexual decision making, whereas socio-economic risk factors were conceptualized into education attainment, employment status and income. The questionnaire was developed in English and translated into Malaysian language by the primary researcher. To ensure consistency, the questionnaire was back-translated into English by a professional translator. Both the English and Malaysian versions of the questionnaires were pre-tested for face validity prior to the survey. Content validity was ensured by a panel of experts, including two public health physicians and one infectious diseases physician. The questionnaire included questions on condom use, socio-demographic characteristics, HIV status and HIV risk-related behaviour.

### Definition of variables

#### Consistent condom use

Participants were asked how often their partners had used condoms when they had had sexual contact, which included vaginal or anal sex during the past six months. The options given were “never”, “occasionally”, “about half the time”, “almost always” and “always”. Due to the importance of consistent condom use for preventing sexually transmitted infections, including HIV, the responses were later dichotomised into “consistent condom users” and “non-consistent condom users”. “Consistent condom users” included those who reported “almost always” and “always” using condoms, while “non-consistent condom users” included those who had never used a condom or only used condoms “occasionally” or “about half the time” [23].

#### Socio-demographic characteristics

A range of socio-demographic characteristics were included in the survey, such as age, ethnicity, religion, study location, relationship status, employment status and education level. The study locations were divided into urban (Federal Territory of Kuala Lumpur, Petaling and Selayang districts) and sub-urban (Hulu Langat and Sepang districts).

#### HIV status

This is based on the self-reported HIV status of the participants and their partners. It was not validated with any biomedical testing or clinic record due to the anonymity of the questionnaire.

#### HIV risk-related behaviour

Participants were asked whether they had been involved in injection drug use or whether they had had multiple sex partners over the past 12 months. Those who had multiple sex partners were asked whether they had received any token in exchange for sex. Participants who answered “yes” were categorized as being involved in sex work.

### Data collection process

During the data collection, participants who consented to participate in the study were requested to complete the survey form after a brief introduction by a trained research assistant. While completing the questionnaire, the participants were free to ask the research assistance for clarification if there was any question or terminology that they did not understand. Once the questionnaire had been completed, it was given to the research assistant in a sealed envelope to ensure confidentiality.

### Data analysis

Data analysis was conducted using STATA Data Analysis and Statistical Software, version 13 [18]. Once the data had been cleaned, descriptive statistical analyses were undertaken to describe the study variables. Further analyses to identify the predictors of consistent condom use were performed, starting with simple logistic regression. This was followed by an adjusted analysis using multivariate logistic regression, where confounding effects of multiple variables towards the outcome measures were suspected. The level of statistical significance (*p*-value) was set at 0.05.

## Results

### General characteristics of participants

The participants’ socio-demographic characteristics, reported HIV status and HIV-related risk behaviour are shown in Table 1.

**Table 1:** Participants’ socio-demography, HIV status and HIV-risk related behaviours (*N* = 221)

Participants’ characteristics	n	%
Marital status		
Married to their current partner	153	69.2
Not married to their current partner	68	30.8
Religion		
Islam	170	76.9
Christianity	7	3.2
Buddhism	12	5.4
Hinduism	32	14.5
Study Location		
Urban	114	51.6
Sub-urban	107	48.4
Age group*		
Youth (≤ 25 years)	13	6.1
Young adults (26–35 years)	65	30.2
Middle-aged adults (36–50 years)	109	50.7
Older adults (≥51 years)	28	13.0
Highest education attainment		
Never attended school or did not finish primary education	21	9.5
Completed primary education	65	29.4
Completed secondary education	122	55.2
Completed tertiary education	13	5.9
Employment status		
Not working	66	29.9
Working part time	58	26.2
Working full time	97	43.9
History of drug use in the last 12 months		
Used injectable drugs	20	9.1
Used non-injectable drugs	67	30.3
Never used any drugs	134	60.6
Had more than one sex partner		
Yes	32	14.5
No	189	85.5
Involved in sex work		
Yes	24	10.9
No	197	89.1
Participants’ HIV status		
Positive	14	6.3
Negative	148	67.0
Unknown	59	26.7
Partners’ HIV status		
Positive	17	7.7
Negative	103	46.6
Unknown	101	45.7
HIV concordance		
Both HIV positive	5	2.3
Sero-discordant (woman +ve, partner -ve or unknown)	9	4.1
Sero-discordant (woman -ve or unknown, partner +ve)	12	5.4
Both HIV negative or unknown	195	88.2

*6 participants did not respond

The majority (69.2%) of the participants were married to their current partner, and 76.9% were Muslim, with an almost equal distribution of participants in urban and sub-urban locations. With respect to education, nearly all (90.5%) participants completed at least primary school. Seventy percent were working, either full or part time. Among the women surveyed, 20 (9.1%) had used injectable drugs in the last 12 months, while 32 (14.5%) had more than one sex partner in the last 12 months. Of these 32 women, 24 (66.7%) reported receiving money, drugs or favours in exchange for sex and were categorized as being involved in sex work. Overall, 6.3% of the survey participants reported being HIV positive, while 7.7% reported having partners who were HIV positive; 26.7% did not know their HIV status, and 45.7% had no information on their partner’s HIV status. One in five women who injected drugs was HIV positive, and 29.2% of sex workers were HIV positive.

In terms of socio-economic characteristics, 70.1% of the participants were working either full time or part time. Twenty-seven participants did not answer the question on household income. Among the 194 participants who responded, their monthly income ranged from RM80 to RM5,000, with a mean (SD) of RM1,460 (RM963) and a median of RM1,200. A total of 63.4% of participants had a household income lower than RM1,500 per month, which marked the poverty line for an average household of four people in Malaysia [24]. Women were the main earner in 38.9% of households, while another 35.8% shared this responsibility with their male partner.

### Prevalence of consistent condom use

The pattern of condom use among the participants is shown in Table 2. The prevalence of consistent condom use in the past 12 months was 19.5% (95% CI: 14.5–25.3%). Slightly more than half (52.5%; 95% CI: 45.7–59.2%) had never used condoms with their partner in their entire relationship. The prevalence of condom use among participants who were not married to their current partner was 38.2%, compared to only 11.1% among participants who were married. Sero-discordant couples reported only 28.6% prevalence of consistent condom use. Additional information describing the reason for condom use, decision making and access to condoms is shown in Table 3.

**Table 2:** Condom use among participants (*N* = 221)

Condom use	*n*	Percentage (%)
Have you ever used a condom?		
Yes	105	47.5
No	116	52.5
Used condoms at last sex?		
Yes	64	28.9
No	41	18.6
Not applicable (never used condom)	116	52.5
Frequency of condom use		
Always (a)	28	12.7
Almost always (b)	15	6.8
About half the time (c)	30	13.6
Occasionally (d)	32	14.5
Never used a condom (e)	116	52.5
Consistent condom user		
Yes (a + b)	43	19.5
No (c + d + e)	178	80.5

**Table 3:** Additional information on condom use among participants who had ever used a condom (*N* = 105)

Additional information on condom use	*n*	Percentage (%)
Reason for using condoms		
To avoid pregnancy	23	21.9
To avoid STIs including HIV	63	60.0
Both of the above reasons	16	15.2
Other reasons	3	2.9
Who decides to use condoms?		
The participant	58	55.2
Her partner	17	16.2
Shared decision by both of them	30	28.6
Who usually provides condoms?*		
Only the participant	74	71.2
Only her partner	18	17.3
Both of them	12	11.5
Where do they normally get their supply of condoms?		
Government clinic	19	18.1
NGOs	52	49.5
Pharmacy	32	30.5
Convenience shop	2	1.9

*One participant did not answer the question

### Predictors of consistent condom use

Further analyses were conducted to identify factors predicting consistent condom use. The findings are shown in Table 4. Variables that showed a *P*-value of less than 0.05 in the simple logistic regression were further analysed using multiple logistic regression. After adjusting for study location, participants’ HIV status and involvement in sex work, the odds of consistent condom use among unmarried women was approximately five times higher than that among women who were married (AOR = 4.95, 95% CI: 2.45, 9.99). Women living in urban areas (AOR = 2.97, 95% CI: 1.30, 6.78), HIV-positive women (AOR = 3.45, 95% CI: 1.13, 10.5) and women who were involved in sex work (AOR = 3.55, 95% CI: 1.45, 8.67) had higher odds of consistent condom use than did their respective reference groups.

**Table 4:** Factors associated with consistent condom use (N = 221)

Socio-demographic characteristics/HIV status/HIV risk behaviour	Consistent condom use				Multiple logistic regression	
	Yes (*n* = 43)		No (*n* = 178)			
	*n*	%	*n*	%	*P*-value	Adjusted odds ratio ^a^ (95% CI)
Marital status						
Married to the current partner	17	11.1	136	88.9	< 0.001*	Ref
Not married to the current partner	26	38.2	42	61.8		4.95 (2.45, 9.99)
Religion						
Muslim	29	17.1	141	82.9	0.10	
Non-Muslim	14	27.5	37	72.5		
Study Location						
Urban	34	42.5	80	57.5	0.007*	2.97 (1.30, 6.78)
Sub-urban	9	9.2	98	90.8		Ref
Age group						
Youth (≤ 25 years)	0	0	13	100.0	0.34	
Young adults (26–35 years)	12	18.5	53	81.5		
Middle-aged adults (36–50 years)	22	20.2	87	79.8		
Older adults (≥51 years)	6	21.4	22	78.6		
Highest education attainment						
Primary education or less	21	24.4	65	75.6	0.14	
Completed at least secondary education	22	16.3	113	83.7		
Employment status						
Working	32	20.6	123	79.4	0.49	
Not working	11	16.7	55	83.3		
Participants’ HIV status						
Positive	6	42.9	8	57.1	0.04*	3.45 (1.13, 10.5)
Negative or unknown	37	17.9	170	82.1		Ref
Partners’ HIV status						
Positive	6	35.3	11	64.7	0.11	
Negative or unknown	37	18.1	167	81.9		
Used injectable drugs						
Yes	3	15.0	17	85.0	0.77	
No	40	19.9	161	80.1		
Involved in sex work						
Yes	10	41.7	14	58.3	0.004*	3.55 (1.45, 8.67)
No	33	16.8	164	83.2		Ref

* Significant at *P* < 0.05 ^a^ Adjusted odds ratio from multiple logistic regression adjusting for marital status, study location, participants’ HIV status, and involvement in sex work Ref = reference group in logistic regression

## Discussion

A key finding of this study is the high prevalence of unprotected sex among the participating women, whereby only one in five women had used condoms regularly with their partners during the 12 months prior to the study. The findings of this study resonate with previous research findings on condom use among MWIDs in Malaysia, which ranged from 14 to 22% in studies conducted in Kuala Lumpur and five other cities in Peninsular Malaysia [2, 8, 9]. However, data from these studies included condom use for all sexual contacts of MWIDs, regardless of whether the contact occurred with their long-term steady partners or with casual partners. Therefore, the results of the present study provide a more specific prevalence of consistent condom use within the intimate relationships of MWIDs and their long-term partners. This is important to distinguish because the issues that affect condom use and intervention plans for promoting safer sexual practices varied according to the type of relationship [3, 25]. This finding was elaborated upon by Diaz-Loving and Villagran-Vazquez (1999) in their work on the determinants of behavioural changes in the context of heterosexual HIV prevention in Mexico [26]. The researchers construed that subjective norms and the motivation for complying with reference groups appeared to be important determinants of condom use among women with regular partners.

In this study, a significantly lower proportion of condom users were married women and those living in rural areas, which could be due to the stronger effect of socio-cultural factors and gender norms on these groups [27]. As an example, the norms of an ideal family life with both parents available to raise their children together led many women to remain married as they tried to fit into the expected role of a wife and mother. Being married also meant that the women were fully committed to the relationship and bound to the expected roles of a wife. In contrast, these expectations were not present among the unmarried couples, as the nature of their relationship is non-traditional in Malaysian culture. This is consistent with the finding of more consistent condom use among the unmarried women. In addition to their greater ability to make sexual decisions, unmarried women were not restricted by socio-cultural expectations in a marriage and therefore faced fewer barriers to sexual communication.

Condoms were also not popular among the youth, as none of the women aged 25 years and younger reported high condom use. The theory of gender and power suggests that power imbalances in a relationship can affect women’s ability to negotiate for safer sexual practices [28]. Previous research has proposed that Malaysian women prefer to discuss issues with their partners before making decisions related to their own sexual and reproductive health [29]. Additionally, their decision-making abilities were strongly influenced by socio-cultural norms that served to entrench gender role expectations and enhanced their inferior position within the relationship. This situation is reported to be common in the patriarchal society of Malaysia [11, 27]. The implications of the participants’ compliance with gender norms reduced the autonomy they had over their own bodies.

The results revealed a high proportion of unprotected sex among sero-discordant couples, whereby only approximately one in four women reported consistent condom use despite being aware that their partners were HIV positive. It was also noted that nearly half of the women did not know of their partners’ HIV status. This put the women whose partners had HIV at great risk of being infected. A study conducted among Indian women that examined the factors associated with unprotected sex revealed a similar finding [30]. In that study, concerns regarding a partner’s trust were identified as important factors contributing to women’s lack of condom use despite their knowledge of the HIV-positive status of their sexual partners.

Several limitations were identified with the performance of this study. The use of RDS led to non-probability sampling in the survey, which limited the generalizability of the survey results [31] and introduced selection bias into the sample [32]. Furthermore, this was the first time that RDS had been used to recruit the partners of MWIDs, although the technique had previously been reported as useful for recruitment within a community of men who injected drugs [33, 34]. Although RDS is believed to be the best way to obtain a representative sampling of a hidden population, it proved extremely difficult to sustain the referral chain to reach the women because the referrals mainly depended on the network of MWIDs. There were not enough waves of chain referrals in several locations, as the network of MWIDs tends to be very small and mobile. This is due to their injecting habits; they preferred to inject in smaller groups and in a secluded and secure place as a survival strategy against frequent police raids. The tight law enforcement by police has led people who inject drugs (PWIDs) to make only brief contact with each other, essentially for obtaining their drug supply. This has made it difficult for them to recruit their peers into this study. Another limitation is related to the nature of the data collection, which is self-reported and may introduce information bias. The cross-sectional design also restricted any interpretations of causal relationships in the study findings.

## Conclusion

The results from this study have shown that women who are intimately involved with PWIDs are vulnerable to HIV. Unprotected sex was a common practice, with only one in five women using condoms regularly with their partners. The high prevalence of HIV among their partners and the low use of condoms within their relationships underscore the heightened risk faced by these women. The challenges of consistent condom use within a long-term relationship call for other preventive strategies of HIV prevention within this population. These strategies need to include strengthening the HIV screening of PWIDs and encouraging their disclosure of their HIV status to their partners, while simultaneously empowering women by providing alternative prevention methods that they are able to control.

## Data Availability

The datasets used and analysed during the current study are available from the corresponding author on reasonable request.

## Abbreviations

AOR: Adjusted odds ratio
HIV: Human immunodeficiency virus
MWID: Man who injects drugs
NGO: Non-government organizations
PWID: Person who injects drugs
